# Cystic Disease of the Antrum

**Published:** 1897-12

**Authors:** J. L. Clark

**Affiliations:** Stratford, Canada


### CYSTIC DISEASE OF THE ANTRUM.

By J. L. Clark, V.S.,

STRATFORD, CANADA.

*Subject:* Bay gelding, two years old, blooded. Symptoms, enlarged condition of frontal and superior maxillary sinus upon left side of face (the frontal presenting the most prominent appearance midway between the eye and the median line). Difficult breathing, watery discharge from the left eye, muco-purulent discharge from corresponding nostril, of fetid odor. Upon percussion, a hollow sound was emitted.

*Treatment.* Animal cast in usual manner; a longitudinal incision was made upon the most prominent eminence; the periosteum was carefully divided, a blunt knife used to separate it from the bone, the trephine then being used; after removing the bone a well-filled sac of a bluish-gray color protruded through the opening; after puncturing and evacuating a portion of the fluid it was deemed necessary to make another opening at the lower border of the sinus, which was done, so as to allow drainage, the sac again being punctured, from which came a muco-serous discharge containing numerous large blood-clots.

The sinus was then carefully scraped with a bone-spoon, and all foreign substance removed; the superior incision was carefully drawn together and sutured, the inferior opening plugged with a pledget of tow dressed with the following injection: Alum pul., ℥ss; tr. iodine, ℥ij; aqua, Oj; one tablespoonful of the above solution in a cup of distilled water injected into sinus twice daily.

After treatment for four weeks the animal made a good recovery, and to-day is all right, the only visible signs being two small longitudinal incisions.

